# A Retrospective Study of the Incidence of Bacterial Sexually Transmitted Infection (Chlamydia and Gonorrhea) in the Mississippi Delta Before and During the COVID-19 Pandemic

**DOI:** 10.7759/cureus.23712

**Published:** 2022-03-31

**Authors:** Maria L Ozua, Al Artaman

**Affiliations:** 1 Family Medicine, Oceania University of Medicine, Apia, WSM; 2 Research, Oceania University of Medicine, Apia, WSM

**Keywords:** sexually transmitted infection (sti), mississippi delta, gonorrhea, covid-19, chlamydia

## Abstract

Background: Sexually transmitted infections (STIs), such as gonorrhea and chlamydia infections, are prevalent worldwide, in the United States, and in Mississippi (MS). The COVID-19 pandemic has impacted the healthcare system, particularly in disadvantaged areas such as the MS Delta.

Methods: A retrospective analysis of medical records of three clinics in the MS Delta was conducted during pre-COVID-19 (July 2019 to September 2019) and in the same months (July 2020 to September 2020) during COVID-19 in 2020. Patients tested for STIs were identified using infection diagnosis codes. We calculated percentages and means for demographic variables, changes between the two years, and computed the percentage of patients who tested positive for each year.

Results: Out of the 25 patients tested for STIs, 11 were tested in 2019 and 14 in 2020. Among those tested in 2019 compared to 2020, patients were younger (average age: 34.3 years in 2019 vs. 29.6 years in 2020), had a larger percentage of females (81.2% in 2019 vs. 50.0% in 2020) and African Americans (72.7% in 2019 vs. 57.1% in 2020), and more were uninsured (27.3% in 2019 vs. 42.9% in 2020). Of the three clinics, clinic #1 tested most patients (72.7% in 2019 vs. 64.3% in 2020). Among the tested patients, 0% had confirmed positive results in 2019 and 21.4% in 2020. The incidence of positive results in 2020 was 7.1% for chlamydia and 14.3% for gonorrhea.

Conclusion: The incidence of gonorrhea and chlamydia increased during COVID-19 in 2020. So, it is of paramount importance to encourage increased testing and targeted interventions for high-risk groups for STIs.

## Introduction

Sexually transmitted infections (STIs) are prevalent worldwide with increasing incidence rates. More than one million STIs such as chlamydia, gonorrhea, syphilis, trichomoniasis, herpes simplex virus, and human papillomavirus are acquired every day. There are an estimated 376 million new infections with one of the four STIs (i.e., chlamydia, gonorrhea, syphilis, and trichomoniasis) each year [1,2]. STIs are a growing concern worldwide and in the United States. In addition, it is estimated that medical costs of 16 billion dollars may be attributed to STIs. It is important to understand the testing and treatment of STIs, especially chlamydia and gonorrhea [2].

STIs can be spread from one person to another through sexual contact and can be caused by various organisms, i.e., bacteria, viruses, and parasites. Bacteria cause chlamydia, gonorrhea, and syphilis. STIs caused by viruses include HIV/AIDS, herpes simplex virus, and the human papillomavirus. Parasites, such as *Trichomonas vaginalis*, can cause STIs such as trichomoniasis [3]. The Centers for Disease Control and Prevention (CDC) track notifiable STIs, which include chlamydia and gonorrhea [4]. Healthcare providers share information on the case identification and testing of patients with chlamydia and gonorrhea with the CDC [4]. This reporting can help the government track these STIs to assist in the understanding of bacterial STIs and suggest ways to enhance prevention and treatment measures to effectively reduce the transmission of these infections. It is imperative to understand the epidemiology and incidence, including testing and treatment of chlamydia and gonorrhea at local, state, and national levels.

In 2019, the CDC reported that more than 2.4 million cases of chlamydia and gonorrhea were reported from all states and territories [5]. Like other states in the deep south, Mississippi (MS) has disproportionately high rates of STIs and HIV, especially among racial and sexual minorities [6-8]. By 2019, MS was one of the states where the incidence of STIs was high, and the state had a high number of reported rates per 100,000 population for both chlamydia and gonorrhea [5]. MS has an estimated population of 2,961,279 as of April 1, 2020. Among all residents, 59.1% were Whites, 37.8% were Blacks, 3.4% were Hispanics or Latinos, and 1.1% were Asians [7]. The median household income from 2015 to 2019 was $45,081. The poverty rate of MS was 19.6%, compared to a poverty rate of 11.4% in the United States [7].

In the northwestern section of MS, the MS Delta encompasses almost 7,000 square miles of alluvial floodplains. MS has 82 counties, including 18 counties that are in the MS Delta where agriculture has been the mainstay of the economy. Currently, 15 counties in the MS Delta have a population of Blacks or African Americans, with per capita income that ranges between $13,924 and $29,190. Overall, rural Mississippians have poorer health, higher poverty rates, an insufficient supply of medical care providers, and a cultural climate that likely contributes to the spread of STIs [8]. In 2018, Barger et al. reported that “people living in the Delta Region of MS face significant disparities in the incidence of chlamydia, gonorrhea, and syphilis - in many instances a near-doubling of risk. Double disparity consists of a service population experiencing disproportionately high rates of risky health behaviors coupled with historically low funding and investment” [9]. The prevalence of chlamydia and gonorrhea among young adults in MS suggests an urgent need to examine the data to better understand the factors that contribute to the high percentage of the STIs and develop methods to reduce the incidence of these STIs in MS and the MS Delta [9].

Chlamydia is caused by the bacterium *Chlamydia trachomatis* and is the most common STI in the United States [10]. Of all cases of reported chlamydia in the state of MS, 23.6% were reported from the MS Delta [5]. Nationally and statewide, women had higher rates of chlamydia compared to men [11,12]. In 2018, young adults in the 20-24 age group had the highest incidence rate of 4,068.5 per 100,000 population. This was followed by young persons in the 15-19 age group with a rate of 3,493.3 per 100,000 population [5]. The number of chlamydia cases has increased over time, especially among the younger age groups, i.e., 20-24 years in MS [5].

There are stark differences in the rate of chlamydia among racial and ethnic groups. Blacks have the highest reported number of cases at both the national and MS levels compared to other racial or ethnic groups. In 2018, there were 484,785 cases (or a rate of 1186.4 per 100,000 population) reported in the United States among Blacks. Among these cases, MS reported 12,031 cases (or a rate of 1078.1 per 100,000 population). During the same year, Whites had 419,627 cases (or a rate of 212.1 per 100,000 population) in the United States and 2,695 cases (or a rate of 160.3 per 100,000 population) in MS [5]. Minimal numbers of chlamydia were reported in other racial or ethnic groups in the MS during the same period [5,11].

The common symptoms of chlamydia include painful urination, discharge from the penis or vagina, painful sexual intercourse, bleeding between periods in women, and testicular pain in men. Chlamydia can also infect the rectum with rectal pain, discharge, bleeding, or no signs [12,13]. If left untreated, chlamydia can cause cervicitis, pelvic inflammatory disease, infertility, chronic pelvic pain, and ectopic pregnancy [14].

Screening recommendations and risk factors have been identified for chlamydia. The CDC recommends chlamydia screening for sexually active women of 25 years or younger, pregnant women, and both women and men in risk groups [12,14]. The common risk factors for chlamydia include having unprotected sex, multiple sexual partners, and men having sex with men [6,12,15].

Gonorrhea is caused by the bacterium *Neisseria gonorrhoeae *that grows in the reproductive tract of women and the urinary tract of both men and women [14]. In 2019, a total of 616,392 cases of gonorrhea were reported to the CDC, making it the “second-most common notifiable” condition in the United States for that year [16]. Rates of reported gonorrhea have increased 92.0% since the historic low in 2009. During 2018-2019, the overall rate of reported gonorrhea increased 5.7% [17].

In 2019, the CDC reported that among all states, Mississippi ranked #2 in rate/100,000 population, 12,068 (or a rate of 405.5 per 100,000 population) [17]. Approximately, 23% of these infections were reported from the MS Delta areas [5,11]. In Mississippi, there were slightly more women with 4,888 cases compared to 4,846 cases of men with gonorrhea infections [5,11]. Overall, younger adults were most likely to have the highest risks of contracting gonorrhea [18,19]. In the United States, younger adults in the 20-24-year age group had the highest number of cases in the United States, with 157,708 cases (or a rate of 3,275 per 100,000 population) [5]. The age group with the second-highest number of reported cases were those in the 25-29-year group with 129,385 cases [5]. In MS, individuals in the 15-19-year age group had the second-highest number with 2,202 cases (or a rate of 1067.4/100,000 population), followed by the 25-29-year age group with 2,032 cases [5]. This trend was linked to the increasing number of young individuals who were engaging in risky sexual behaviors [18-20]. Lower numbers were reported in older adults, but Bowen et al. revealed that gonorrhea infections have been increasing from 2013 in all age groups [21].

Gonorrhea symptoms in men include having a burning sensation when urinating or a discharge from the urethra. Women can have a mild course but can lead to pelvic inflammatory disease, with a risk of infertility. Mother to the fetal transmission can occur during labor and delivery. Gonorrhea can spread to the blood or joints and become life-threatening if left untreated [11,14].

Individuals who are infected with gonorrhea are more likely to get other STIs compared to individuals who are gonorrhea-free. This may be attributed to the fact that some of the behaviors and circumstances that may put these individuals at the risk for gonorrhea can also put them at greater risk for contracting other STIs such as chlamydia and HIV [16,21]. Although reported cases decreased from 2008 to 2009 and 2012 to 2013, they have steadily been increasing since 2013. Other studies have shown that African American women reported greater substance abuse, more sexual partners, higher concurrency levels, and more transactional sex and may be at higher risk than the females of other racial/ethnic groups [16].

The severe acute respiratory syndrome coronavirus 2 (SARS-CoV-2) started spreading in the United States in January 2020 and was declared a pandemic in March 2020 [22]. The global coronavirus disease or COVID-19 pandemic has massively affected societies, the economy, the healthcare system, particularly the healthcare infrastructure [9,22-25]. Major efforts were employed to contain the spread of the disease, including sealing borders for international travels, wearing masks, practicing social distancing, etc. [22,24]. By July 1, 2020, the number of COVID-19 cases in Mississippi was 28,770 and increased to 493,670 cases by September 30, 2020 [24]. CDC reported that during the early months of the COVID-19, pandemic reports of STIs decreased due to disruptions in the healthcare system and STI testing services [23,24].

Testing and treatment of these STIs are critical nationally and in states with high rates of STIs. The purpose of this retrospective epidemiological study is to describe and compare the incidence of gonorrhea and chlamydia in primary medical clinics in the Mississippi Delta during two periods. The study periods compared were pre-COVID-19 (July 2019 to September 2019) and during COVID-19 (July 2020 to September 2020). This research study will examine and compare STI testing and confirmed STI cases for a sample of patients treated for chlamydia and gonorrhea in three clinics in the Mississippi Delta area during the two study periods.

The present research was guided by two research questions: (1) What were the percentages of gonorrhea and chlamydia among people screened for STIs during the two study periods in the Mississippi Delta? (2) How did the incidence of confirmed STI test results among tested individuals change during the two study periods in the Mississippi Delta?

This study is expected to estimate the magnitude of changes in the percentage of confirmed STI cases in the communities of the Mississippi Delta area. The hypothesis of this study is that the number and percentage of confirmed STI results during the COVID-19 period will be greater than those found during the pre-COVID-19 period in the communities of the Mississippi Delta area. Local authorities can use the information to understand if the epidemiology of STIs under analysis is changing. This information could be beneficial to recommend prevention programs that would help to control the disease transmissions.

## Materials and methods

Data sources

This retrospective review of the electronic medical records of patients with diagnoses of chlamydia or gonorrhea was completed in three well-established primary medical clinics located in the heart of the agricultural Mississippi Delta. These three clinics are owned by the same company. Each clinic has one to two nurse practitioners. Two full-time physicians examined and treated patients in all three clinics. As the clinics are using electronic medical records, data were obtained from the main office that was located away from these clinics.

All clinics were in counties within the Mississippi Delta area. The population size of each of the counties varied across these agricultural areas. The demographic and economic characteristics of persons residing in these counties varied vastly across the three counties. Appendix A provides an overview of several basic demographic and economic characteristics of these counties in which each of the three clinics is located.

At the central office, a senior office staff member extracted the data from medical records for the study. The Current Procedural Terminology (CPT®) codes, maintained by American Medical Association, were used to select the STIs of interest from the medical records. The codes used were all under the range "Infectious Agent Antigen Detection." We used the following CPT codes: 87591 (*Neisseria gonorrhoeae*) and 87491 (*Chlamydia trachomatis*). Using these codes, records were abstracted for all patients who were tested and diagnosed for STIs. Each subject was given a unique code that identifies them for this study.

To assess the changes between the two time periods, data were collected on STI screening of all patients who visited the clinics in pre-COVID-19 and during COVID-19 periods. The dates were July 2019 through September 2019 (pre-COVID-19) and July 2020 through September 2020 (during COVID-19).

Inclusion and exclusion criteria

The inclusion criteria include Mississippi residents that attended one of the three clinics in the Mississippi Delta for medical care during the study period: the pre-COVID-19 period and the period during COVID-19. All patients must have been seen and tested for at least one chlamydia or gonorrhea infection at one of the three clinics during the time periods. Patients were excluded from the study if they were not tested for chlamydia and/or gonorrhea. Further, they were excluded if the testing for chlamydia and/or gonorrhea did not occur in at least one of the three study clinics in the Mississippi Delta area during the two study time periods.

Data analysis

The dates and time periods, pre-COVID-19 and during COVID-19, were determined for all patients who were seen at all three clinics in the Mississippi Delta. Descriptive statistics for continuous variables included average, standard deviation, median, and minimum and maximum values of patients who were screened and tested positive for both STIs. For categorical variables, including gender, race/ethnicity, and insurance status, the number and percentages of those who were screened and tested positive were computed for each category of the variables. Additionally, the total number of patients seen and the percentages that tested positive for the STIs in the identified periods were assessed for comparison purposes. We also examined the changes and percentage changes between the pre-COVID-19 and during COVID-19 time periods. MedCalc Statistical Software (MedCalc Software Ltd., Belgium) [26] and Microsoft Excel version 2015 (Microsoft Corporation, New Mexico, USA) were used for the data management and analysis of the data.

Ethics statement

The owner of all three clinics reviewed and approved the proposal for the study. All patient-identifiable information including names, dates of birth, social security numbers, and addresses were not recorded. Confidentiality of all patient data was confirmed before the launch of the study and followed through all aspects of the study. Data obtained were recorded in a Microsoft Excel document and stored on a password-protected laptop.

## Results

The clinics in this study provided clinical and medical services to residents living in three counties located within the Mississippi Delta area. The counties had a diverse population, including individuals of different races and ethnic groups. These groups residing in the counties included 76.5%-96.0% of Blacks or African Americans, 4.0%-22.5% of Whites, 0.0%-0.2% of Hispanics, and 0.3%-0.8% of persons who identified themselves as multiracial. Economic indicators of the counties included 27%-36% of individuals living below poverty and 14%-17% of individuals who were uninsured. Appendix A provides an overview of the basic characteristics of the counties in which each of the three clinics is located for this study.

Among all three clinics, a total of 7,849 patients were seen from July to September 2019 and 7,753 from July to September 2020. Of all the patients seen in the three clinics in 2019-2020, 11 were tested for STIs (i.e., chlamydia and gonorrhea) in 2019, and 14 were tested for STIs in 2020. Of all the three clinics, most patients were seen and tested for STIs in clinic #1. Among all the patients tested for STIs in 2019, 72.7% visited clinic #1, 18.2% visited clinic #2, and 9.1% visited clinic #3. In 2020, the percentage of patients seen and tested for STIs was 64.3% in clinic #1, 35.7% in clinic #2, and 0.0% in clinic #3 (Table 1).

**Table 1:** Number and percent of patients seen and tested for chlamydia and/or gonorrhea in three clinics in the Mississippi Delta during pre-COVID-19 (July-September 2019) and during COVID-19 (July-September 2020) ^1^Number and percentage of patients who visited each of the three clinics during the following dates: July 2019 to September 2019 or July 2020 to September 2020. ^2^Number and percentages of patients who were tested for chlamydia and/or gonorrhea during the following dates: July 2019 to September 2019 or July 2020 to September 2020. STIs: Sexually transmitted infections.

	Patient Visits^1^	STI Tests^2^	Patient Visits^1^	STI Tests^2^	STI Tests^2^
Facility	2019		2020		Total
Clinic #1	4,652	8 (72.7%)	4,358	9 (64.3%)	17 (68.0%)
Clinic #2	638	2 (18.2%)	536	5 (35.7%)	7 (28.0%)
Clinic #3	2,559	1 (9.1%)	2,859	0 (0%)	1 (4.0%)
	7,849	11 (100.0%)	7,753	14 (100.0%)	25 (100.0%)

CPT codes for chlamydia and gonorrhea were used to identify patients who were tested for STIs during the 2019 and 2020 time periods. Patients identified were screened for one or more of the following STIs and infections: chlamydia, gonorrhea, trichomonas, candidiasis, and *Gardnerella vaginalis*. One patient was tested for gonorrhea only, while all other patients were screened for one or more STIs. In 2019, six out of 11 patients were tested for five STIs or infections. In 2020, the majority (eight out of 14) of patients were tested for two STIs (chlamydia and gonorrhea), and no patients were tested for five STIs (Table 2).

**Table 2:** Demographic characteristics of patients who were seen and tested for sexually transmitted infections (STIs) in three clinics in the Mississippi Delta during pre-COVID-19 (July-September 2019) and during COVID-19 (July-September 2020) (N = 25) ^1^Unknown race or ethnic groups include Asians, Hispanics, Whites, and other groups who were recorded as “unknown” in the medical records.

	2019	2020	Change Between Years
	N (%)	N (%)	Change (% Change)
Total	11 (100.0%)	14 (100.0%)	---
Age			
Mean	34.3	29.6	-4.7 (-13.7%)
Standard deviation	11.3	10.2	-1.1 (-9.7%)
Median	30	26	-4 (-13.3%)
Minimum–maximum	24-57	19-49	--
Age groups (in years)			
<19	0 (0.0%)	2 (14.3%)	2 (--)
20-29	5 (45.5%)	6 (42.9%)	1 (20.0%)
30-39	4 (36.4%)	3 (21.4%)	-1 (-25.0%)
40-49	0 (0.0%)	3 (21.4%)	3 (--)
50 years and older	2 (18.2%)	0 (0.0%)	-2 (-100.0%)
Gender			
Female	9 (81.2%)	7 (50.0%)	-2 (-22.2%)
Male	2 (18.2%)	7 (50.0%)	5 (250%)
Race/ethnicity groups			
African American	8 (72.7%)	8 (57.1%)	0 (--)
Unknown^1^	3 (27.3%)	6 (42.9%)	3 (100%)
Had insurance coverage			
Yes	8 (72.7%)	9 (57.1%)	1 (2.5%)
No	3 (27.3%)	6 (42.9%)	3 (100%)

The average age was 34.3 years in 2019 compared to 29.6 years in 2020. In 2019, the median age was 30 years, with a range of 24-57 years compared to 26 years with a range of 19-49 years. Most patients in 2019 were in the 20-29 (45.5%) or 30-39 (36.4%) age groups. In 2020, 42.9% of all patients were in the 20-29 age group. This was followed by the 30-39 (21.4%) and 40-49 (21.4%) age groups.

Females (81.2%) represented the largest percentage of persons who were tested for STIs in the 2019 study period. In 2020, the distribution of males and females was equal to 50% each year. African Americans represented nearly three-fourths (72.7%) of persons tested for STIs in 2019 and 57.1% of those tested for STIs in 2020. More than one out of every four patients (27.3%) who were seen and tested for STIs were uninsured in 2019. The percentage of uninsured patients who were seen and tested at the clinics increased to 42.9% in 2020 (Table 3).

**Table 3:** Number of tests performed for each of the sexually transmitted infections (STIs) in three clinics in the Mississippi Delta during pre-COVID-19 (July-September 2019) and during COVID-19 (July-September 2020) (N = 25) ^1^NG is gonorrhea, caused by the bacterium *Neisseria gonorrhoeae*. ^2^CT is chlamydia, caused by the bacterium *Chlamydia trachomatis*. ^3^TV is trichomonas, a common sexually transmitted disease (STD) that is caused by a protozoan parasite called *Trichomonas vaginalis*. ^4^CA is candidiasis, a fungal infection caused by the organism *Candida albicans*. ^5^GC is *Gardnerella vaginalis*, an infection that can be transmitted from one sexual partner to the other. It can contribute to a common reproductive health condition called bacterial vaginosis.

	NG^1^	NG^1^, CT^2^	NG^1^, CT^2^, TV^3^	NG^1^, CT^2^, TV^3^, CA^4^, GC^5^	Total Tested
	N (%)	N (%)	N (%)	N (%)	N (%)
Clinic #1	0 (0.0%)	2 (40.0%)	0 (0.0%)	6 (100.0%)	8 (72.7%)
Clinic #2	0 (0.0%)	2 (40.0%)	0 (0.0%)	0 (0.0%)	2 (18.2%)
Clinic #3	0 (0.0%)	1 (20.0%)	0 (0.0%)	0 (0.0%)	1 (9.1%)
Total in 2019	0 (0.0%)	5 (100.0%)	0 (0.0%)	6 (100.0%)	11 (100.0%)
Clinic #1	0 (0.0%)	7 (87.5%)	2 (40.0%)	0 (0.0%)	9 (64.3%)
Clinic #2	1 (100.0%)	1 (12.5%)	3 (60.0%)	0 (0.0%)	5 (35.7%)
Clinic #3	0 (0.0%)	0 (0.0%)	0 (0.0%)	0 (0.0%)	0 (0.0%)
Total in 2020	1 (100.0%)	8 (100.0%)	5 (100.0%)	0 (0.0%)	14 (100.0%)

**Figure 1:**
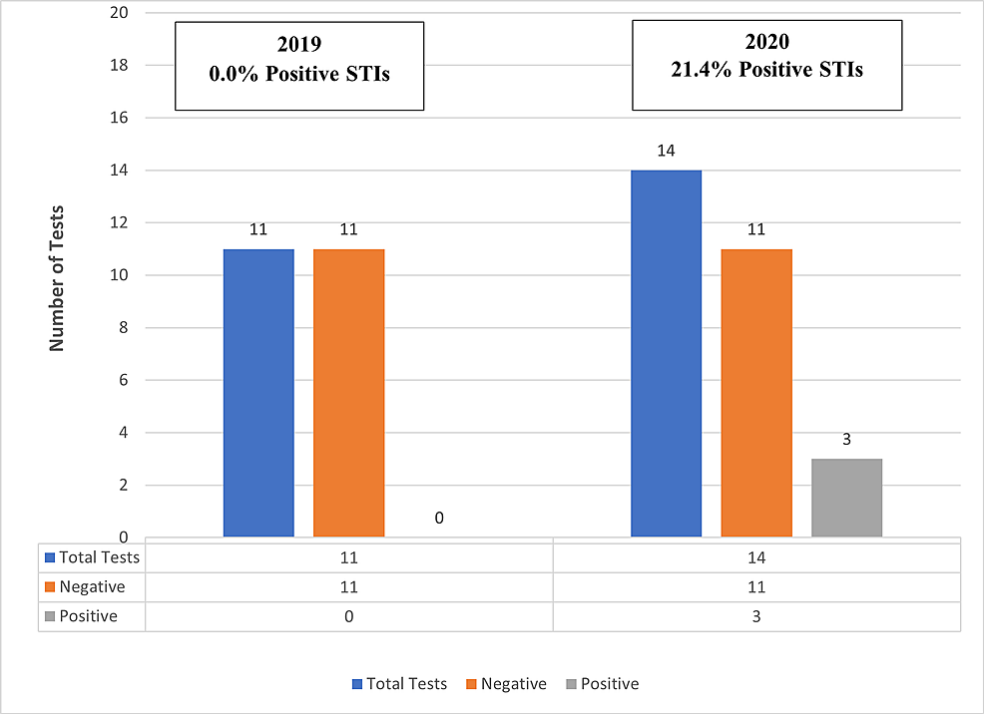
Number and percent of positive test results for all patients who were seen and tested for STIs in 2019 and 2020 at the three clinics in the Mississippi Delta, Mississippi (N = 25) ^1^This is the period from July to September 2019, also referred to as the pre-COVID-19 period. ^2^This is the period from July to September 2020, also referred to as during the COVID-19 period. STIs: Sexually transmitted infections.

Of all patients tested for STIs, only three patients had a positive STI test. In 2019, no persons had a positive test for chlamydia or gonorrhea. In 2019, three out of 14 persons tested positive (21.4%) (Figure 1).

**Figure 2:**
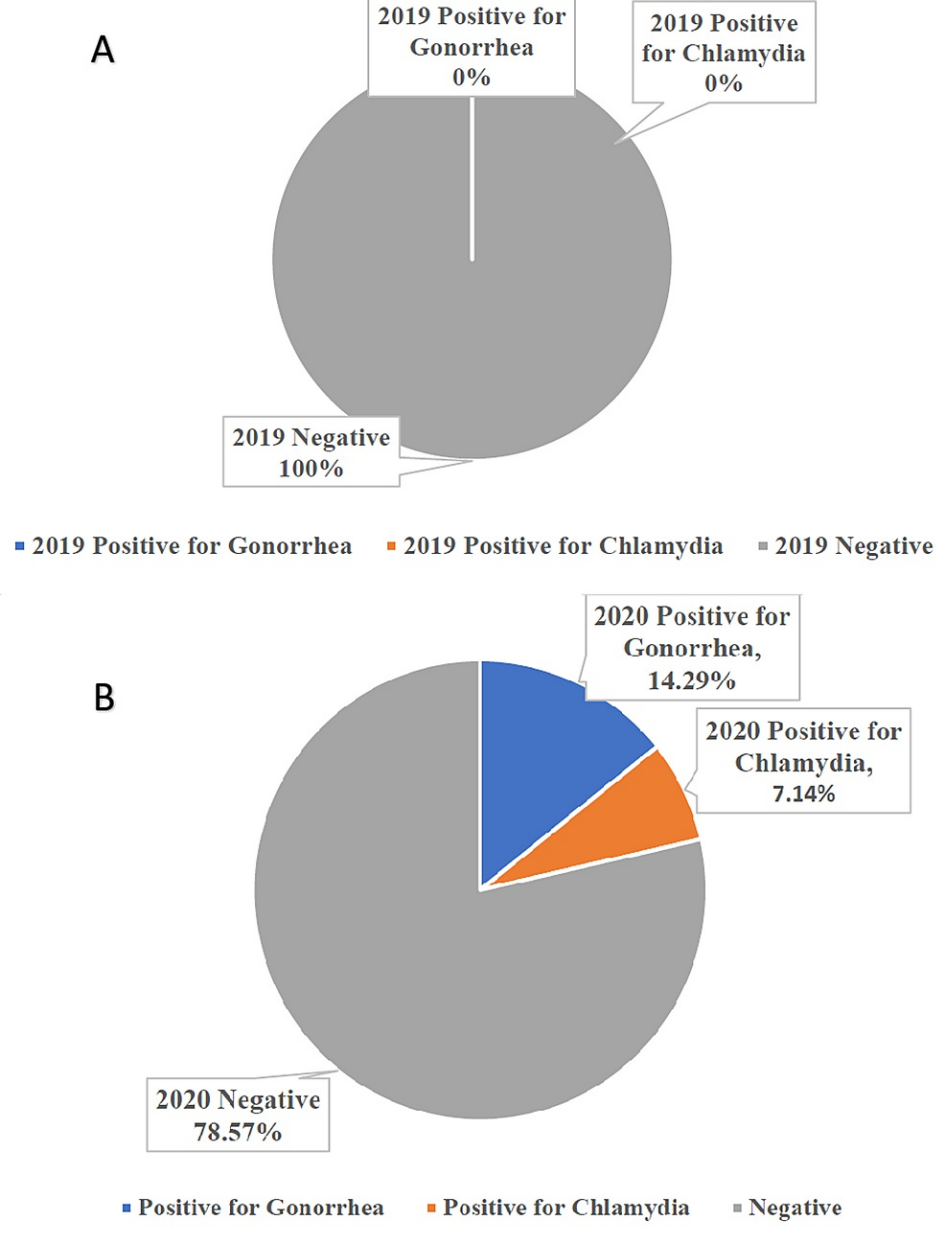
(A) Test results for all patients who were seen and tested for STIs in July-September 2019 at the three clinics in the Mississippi Delta, Mississippi (N = 11). (B) Test results for all patients who were tested for STIs in July-September 2020 at the three clinics in the Mississippi Delta, Mississippi (N = 14) STIs: Sexually transmitted infections.

In 2019, there were no positive tests for gonorrhea or chlamydia out of all 11 tests administered (Figure 2, Panel A). In 2020, there was one positive test for chlamydia and two positive tests for gonorrhea. The incidence rate for positive STIs was 0% in 2019 and 21.4% in 2020. During this year, positivity rates were 7.1% for chlamydia and 14.3% for gonorrhea (Figure 2, Panel B).

## Discussion

This retrospective study assessed the potential impact of the COVID-19 pandemic on bacterial STI testing and confirmed STIs in selected clinics in Mississippi Delta between the pre-COVID-19 period (July-September 2019) and during the COVID-19 period (July-September 2020). The COVID-19 pandemic has impacted the healthcare infrastructure and our access to medical and healthcare services, including how a person may seek and receive care for STIs [23]. There have been multiple measures (i.e., stay-at-home orders, shifts to telemedicine, etc.) and changing recommendations from CDC and other organizations. In May 2020, the CDC provided guidance for sexual health services during the pandemic and “recommended prioritizing patients on the basis of symptoms and risk with routine screening visits deferred until after the emergency response to COVID-19” [23]. Studies of this type that will provide insight and understanding about STI testing in different periods of COVID-19 are of paramount importance.

During the COVID-19 period in our study, the number of patients who were tested and had positive results for bacterial chlamydia and gonorrhea increased compared to the same months in the pre-COVID-19 period. Another study examined testing for bacterial STDs in Maricopa, Arizona, during three periods: pre-lockdown (January-March 2020), lockdown (March-May 2020), and post-lockdown periods (May-December 2020). Similar to this study, Bell et al. reported increases between the lockdown (March-May 2020) and post-lockdown (May-December 2020) periods. However, there were decreases in STI testing between the pre-lockdown and lockdown periods [27]. Another study in California found a decrease in STI testing between the pre-COVID-19 (January-June 2019) and post-COVID-19 (January-June 2020) periods [24]. These studies have reported inconsistent results regarding the impact of COVID-19 on STI testing with different dates for comparisons, varying geographic differences, and differences in demographic compositions. It is imperative that additional studies have to be completed to investigate the potential impact of COVID-19 on STI testing further, especially in areas with high STI rates prior to the COVID-19 pandemic, i.e., the Mississippi Delta area [23,24].

While the cause of the increase in bacterial STIs in the Mississippi Delta reporting during COVID-19 is likely multifactorial, several factors are important to consider. The impacts of COVID-19 were disproportionately felt among various groups, such as age groups, gender, race/ethnicity groups, and the uninsured. Overall, in this study, those tested for STIs were younger, less likely females, and more likely to be uninsured during the COVID-19 period when compared to the pre-COVID-19 period. There were no differences in the number of African Americans who were tested for STIs in the two periods. Johnson et al. reported a decrease in the reporting of STDs among African Americans but no differences by age or sex [24]. Of note, younger individuals and women in the Mississippi Delta area had higher rates of STIs but had smaller percentages of STI testing in 2020 compared to 2019. This phenomenon should be further investigated in the Mississippi Delta to understand the impact of STI testing in the under-resourced Mississippi Delta during COVID-19 [9].

Limitations of the study

This study has several limitations. First, the number of tests completed for STIs in the three clinics in the Mississippi Delta is less during the two study periods: July to September 2019 (pre-COVID-19) and July to September 2020 (during COVID-19). It is possible that the small sample sizes in 2019 and 2020 may have contributed to biased estimates and possibly smaller study reliability. A study has indicated that small sample sizes could negatively affect the precision of estimations [28]. A future study should include more patients, which may decrease the bias and improve the precision of estimates of those who were assessed for STIs during the two time periods.

Second, the data were only collected from three clinics located in the Mississippi Delta area and served the residents of the region. Appendix A shows other medical facilities in the counties in the Mississippi Delta area, and these facilities were not included in the sample. It is possible that patients may have sought medical care from private physicians or other medical facilities other than the three clinics in our sample. A future research study could be expanded to include more clinics and medical facilities in the Delta. This would permit us to increase the sample size and make generalizable statements about our findings to better understand the receipt of STI tests and confirmed STI tests in the Mississippi Delta. Additional sampling techniques such as random selection of clinics or stratification may also be employed in a future sample selection to increase the sample size.

Third, a retrospective study does not permit us to gather data on social factors, such as employment status or behavioral factors, number of sexual partners, drug use, etc. The additional information regarding risk and social factors would enhance our understanding of the incidence of STIs in the Mississippi Delta before and during the COVID-19 pandemic.

Strengths of the study

The study has several strengths. This is one of the few studies that examined testing and the incidence of bacterial STIs in the Mississippi Delta. This study allows clinicians to examine the burden of STIs in the Mississippi Delta area. The study will allow us to gain a better understanding of notifiable bacterial STIs (chlamydia and gonorrhea) in the Mississippi Delta area and the characteristics of these patients. This study can be used to target interventions and community-focused prevention efforts to reduce STIs in Mississippi and the Mississippi Delta area, particularly among young adults.

## Conclusions

Mississippians, especially those residing in the Mississippi Delta area, have a high rate of bacterial STIs, i.e., chlamydia and gonorrhea. The results of the study revealed that there was an increased incidence of gonorrhea and chlamydia in 2020 when compared to the same period in 2019. Future research should aim to address the weaknesses of the present study. In particular, the sample size could be increased to ensure higher reliability of results. This can be achieved by increasing the data collection period and the inclusion of information from more clinics in the analysis. Targeted programs and interventions should be developed to reduce the incidence of STIs in the communities, especially among high-risk groups in the Mississippi Delta area.

## Appendices

Appendix A

**Table 4:** Population and demographic characteristics for each of the study clinics in the Mississippi Delta – July to September 2019 and July to September 2020 ^1^Based on the United States Census Bureau estimates as of April 1, 2020. ^2^Based on the uninsured five-year estimates (2015-2019).

Characteristics	Clinic #1	Clinic #2	Clinic #3
Community population^1^	14,903	1,873	1,299
Race/ethnicity			
% Asians	0.0%	0.0%	0.0%
% Blacks	81.8%	96%	76.5%
% Hispanics	0.2%	0.0%	0.0%
% Multiracial	0.6%	0.3%	0.8%
% Whites	16.7%	4.0%	22.5%
% Poverty rate	35.7%	48.2%	27.2%
Population density (per square mile)	1224	685.8	1012.10
% Uninsured^2^	13.6%	14.3%	17.4%
Availability of additional other healthcare facilities for medical care in the county	1 health department clinic and medical care clinics	1 health department clinic and medical clinics within 20 miles	1 health clinic and medical clinics
Nearby hospitals	One 100+-bed not-for-profit hospital	One 100+-bed hospital within 20 miles	One 100+-bed hospital within 20 miles
